# LeCTR2, a CTR1-like protein kinase from tomato, plays a role in ethylene signalling, development and defence

**DOI:** 10.1111/j.1365-313X.2008.03481.x

**Published:** 2008-04-25

**Authors:** Zhefeng Lin, Lucy Alexander, Rachel Hackett, Don Grierson

**Affiliations:** Plant Sciences Division, School of Biosciences, University of Nottingham, Sutton Bonington CampusLoughborough LE12 5RD, UK

**Keywords:** LeCTR2, ethylene signalling, protein–protein interaction, protein kinase, defence, tomato

## Abstract

Arabidopsis AtCTR1 is a Raf-like protein kinase that interacts with ETR1 and ERS and negatively regulates ethylene responses. In tomato, several CTR1-like proteins could perform this role. We have characterized LeCTR2, which has similarity to AtCTR1 and also to EDR1, a CTR1-like Arabidopsis protein involved in defence and stress responses. Protein–protein interactions between LeCTR2 and six tomato ethylene receptors indicated that LeCTR2 interacts preferentially with the subfamily I ETR1-type ethylene receptors LeETR1 and LeETR2, but not the NR receptor or the subfamily II receptors LeETR4, LeETR5 and LeETR6. The C-terminus of LeCTR2 possesses serine/threonine kinase activity and is capable of auto-phosphorylation and phosphorylation of myelin basic protein *in vitro*. Overexpression of the LeCTR2 N-terminus in tomato resulted in altered growth habit, including reduced stature, loss of apical dominance, highly branched inflorescences and fruit trusses, indeterminate shoots in place of determinate flowers, and prolific adventitious shoot development from the rachis or rachillae of the leaves. Expression of the ethylene-responsive genes *E4* and *chitinase B* was upregulated in transgenic plants, but ethylene production and the level of mRNA for the ethylene biosynthetic gene *ACO1* was unaffected. The leaves and fruit of transgenic plants also displayed enhanced susceptibility to infection by the fungal pathogen *Botrytis cinerea*, which was associated with much stronger induction of pathogenesis-related genes such as *PR1b1* and *chitinase B* compared with the wild-type. The results suggest that LeCTR2 plays a role in ethylene signalling, development and defence, probably through its interactions with the ETR1-type ethylene receptors of subfamily I.

## Introduction

Ethylene regulates many aspects of plant development and responses to biotic and abiotic stress. Perception of ethylene in Arabidopsis is achieved by five members of a family of ER membrane-bound receptors: ETR1, ETR2, ERS1, ERS2 and ethylene insensitive 4 (EIN4), some of which have histidine kinase activity (Chang *et al.*, 1993; Hua *et al.*, 1995, 1998; Sakai *et al.*, 1998; Wang *et al.*, 2002). Although similar, the ethylene receptors can be divided into two subfamilies based on phylogenetic analysis and some shared structural features, with subfamily I being composed of ETR1 and ERS1, and subfamily II being composed of ETR2, ERS2 and EIN4 (reviewed by Hall *et al.*, 2007). Signal transmission involves the downstream Raf-like protein kinase AtCTR1, which negatively regulates ethylene responses (Kieber *et al.*, 1993). AtCTR1 possesses serine/threonine kinase activity, with enzymatic properties similar to those of Raf-1 (Huang *et al.*, 2003). The AtCTR1 N-terminus requires a critical Gly354 residue for interactions with the subfamily I ethylene receptors, and mutation of this residue abolishes the interaction with the receptors (Clarke *et al.*, 1998; Huang *et al.*, 2003). The *ctr1* loss-of-function mutant displays characteristic constitutive ethylene responses in the absence of ethylene, such as the triple response of dark-grown seedlings (Kieber *et al.*, 1993). Transgenic Arabidopsis overexpressing the AtCTR1 N-terminus display constitutive ethylene response phenotypes, whereas AtCTR1 in which the Gly354 N-terminus has been mutated to Glu has no effect, suggesting that the truncated wild-type AtCTR1 N-terminus competes with full-length AtCTR1 for binding to the receptor *in vivo* (Huang *et al.*, 2003).

*EDR1* (*enhanced disease resistance 1*) encodes a kinase with similarity to AtCTR1 (Frye and Innes, 1998) that is involved in disease resistance. *edr1* mutant plants, which have a C→G conversion at nucleotide 1235 that generates an early stop codon, are resistant to powdery mildew caused by the fungus *Erysiphe cichoracearum* (Frye and Innes, 1998). Dark-grown seedlings of the *edr1* mutant show no characteristics of the triple response, but the mutant senesces early in response to ethylene treatment (Frye and Innes, 1998; Frye *et al.*, 2001) and displays enhanced stress responses and spontaneous necrotic lesions under drought conditions in the absence of pathogen. It has been suggested that EDR1 functions at a point of cross-talk between ethylene and salicylic acid signalling (Tang *et al.*, 2005), although it is not known whether EDR1 has any direct associations with ethylene receptors.

In tomato, ethylene perception is more complicated than in Arabidopsis, with six putative ethylene receptor genes (*LeETR* s) and four *AtCTR1-like* genes (*LeCTR* s). *LeETR1* (Lashbrook *et al.*, 1998; Zhou *et al.*, 1996a), *LeETR2* (Lashbrook *et al.*, 1998; Zhou *et al.*, 1996b) and *Never ripe* (*Nr*) (Wilkinson *et al.*, 1995) belong to subfamily I, whereas *LeETR4*, *LeETR5* (Tieman and Klee, 1999) and *LeETR6* (Ciardi and Klee, 2001) belong to subfamily II (Klee and Tieman, 2002). The receptors are expressed in various temporal and spatial patterns, depending on developmental stage and external stimuli (Lashbrook *et al.*, 1998; Payton *et al.*, 1996; Tieman and Klee, 1999). *LeETR1*, for example, is expressed constantly in all tissue examined and shows no induction by exogenous ethylene, whereas NR expression increases during ripening, senescence and abscission (Lashbrook *et al.*, 1998; Payton *et al.*, 1996). The *AtCTR1-like* genes in tomato include *LeCTR1*, *LeCTR2*, *LeCTR3*, *LeCTR4* and the *LeCTR4* splicing variants *LeCTR4sv1* and *LeCTR4sv2* (Adams-Phillips *et al.*, 2004; Lin *et al.*, 1998). *LeCTR3* fully complemented the *ctr1-8* mutation, and *LeCTR1* and *LeCTR4* partially complemented it, suggesting that several CTRs may mediate ethylene signalling in tomato (Adams-Phillips *et al.*, 2004). *LeCTR1* is reported to respond rapidly to exogenous ethylene, whereas *LeCTR3*, *LeCTR4* and *LeCTR4sv* mRNAs showed no significant accumulation in response to the hormone.

*LeCTR2*, which we isolated previously (Lin *et al.*, 1998) and called *TCTR2*, encodes a AtCTR1-like kinase. We report here that its N-terminus selectively interacts with a subset of ethylene receptors, and its C-terminus possesses kinase activity. Transgenic tomato plants overexpressing the LeCTR2 N-terminus display altered growth habit, increased ethylene responses, and enhanced susceptibility to the fungal pathogen *Botrytis cinerea.* These results indicate that LeCTR2 plays a direct role in ethylene and defence signalling through its interactions with a subset of ethylene receptors.

## Results

### Sequence and expression of LeCTR2

The 12 kb *LeCTR2* coding sequence consists of 13 exons and 12 introns (Lin *et al.*, 1998). The *LeCTR2* coding cDNA is 2949 bp in length and encodes a 982 amino acid protein (accession number AJ318955). The C-terminus (amino acids 683–982) contains a highly conserved kinase domain, which includes a protein kinase ATP-binding signature (amino acids 714–736: IGLGSYGEVYhAdwngtev), a serine/threonine protein kinase active site signature (amino acids 827–839: IvHrDLKspNLLV), and 11 protein kinase sub-domains (data not shown). The N-terminus contains the conserved motifs (CN box) that exist in all CTR1-like proteins (Huang *et al.*, 2003). Overall, *LeCTR2* is more similar to *EDR1* than to the other *LeCTR* s. *LeCTR2* has the same number, size and position of exons as *EDR1* (Figure S1a) and the two proteins share 65% similarity across their entire sequences (data not shown). This gene structure contrasts with that of other *LeCTR* s; *LeCTR1* and *LeCTR4* have 15 exons with similar size and position, whereas *LeCTR3* and *AtCTR1* have 16 exons (Adams-Phillips *et al.*, 2004). Phylogenetic analysis also indicated that *LeCTR2* and *EDR1* appeared in a distinct cluster from other *LeCTR* s and *AtCTR1* (Figure S1b).

**Figure 1:**
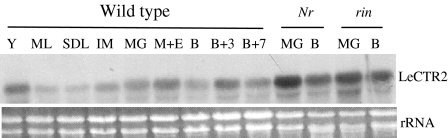
Expression of *LeCTR2* in various organs and at various stages of development. Northern analysis was carried out using 30 μg total RNA. Y, young leaves; ML, fully expanded mature leaves; SDL, seedlings; IM, immature fruit; MG, mature green fruit; M + E, mature green fruit treated with 10 ppm exogenous ethylene for 6 h; B, fruit at onset of ripening (breaker); B + 3, fruit at 3 days post-breaker; B + 7, fruit at 7 days post-breaker; *Nr*, *Never-ripe* mutant; *rin*, *ripening inhibitor* mutant. The *LeCTR2* cDNA (nucleotides 151–950) was used as a probe, and RNA loadings are indicated by the ethidium bromide-stained rRNA gel.

*LeCTR2* mRNA was abundant in young leaves and developing and ripening fruit compared with either seedlings or fully expanded leaves. There was a small increase in expression following treatment of mature green fruit with exogenous ethylene (Figure 1), but expression was highest in fruit of the transcription factor ripening mutant *ripening inhibitor* (*rin*) and the ethylene-insensitive mutant *Never-ripe* (*Nr*) compared with wild-type.

### Interactions of LeCTR2 with LeETR1 and LeETR2

**Figure 2:**
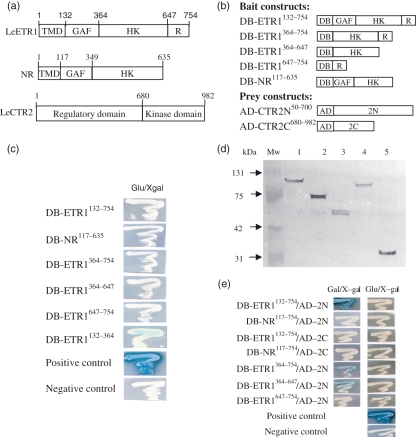
Interaction assays of LeCTR2 with LeETR1 and NR in the yeast two-hybrid system. (a) Structures of LeETR1, NR and LeCTR2, with residues numbered. (b) Constructs used for protein–protein interaction assays in yeast. (c) Yeast expressing bait proteins in the absence of the prey constructs was tested for activation of the *LacZ* reporter gene by incubation on minimal medium containing glucose (Glu) and X-gal (5-bromo-4-chloro-3-indolyl-β-D-galactopyranoside). (d) Synthesis of bait proteins in yeast was detected by Western blotting using an anti-LexA antibody. Lane 1, DB–ETR1^132–754^; lane 2, DB–ETR1HKR^364–754^; lane 3, DB–ETR1HK^364–647^; lane 4, DB–NR^117–635^; lane 5, DB–ETR1R^647–754^. Mw, molecular weight markers. (e) Yeast expressing DB–ETR1^132–754^/AD–CTR2N^50–700^, DB–NR^117–635^/AD–CTR2N^50–700^, DB–ETR1^132–754^/AD–CTR2C^680–982^, DB–NR^117–635^/AD–CTR2C^680–982^, DB–ETR1HKR^364–754^/AD–CTR2N^50–700^, DB–ETR1HK^364–647^/AD–CTR2N^50–700^ or DB–ETR1R^647–754^/AD–CTR2N^50–700^ was tested for *LacZ* reporter gene expression on minimal medium containing X-gal in the presence of galactose (Gal/X-gal) or glucose (Glu/X-gal).

To test whether LeCTR2 was able to interact with ethylene receptors, two subfamily I receptors, LeETR1 and NR, were tested for interaction in the LexA-based yeast two-hybrid system described by Golemis and Brent (1997) (see Experimental procedures). Various regions of *LeETR1* cDNAs (Figure 2a), encoding the receptor without the transmembrane domain (ETR1^132–754^), the GAF domain (ETR1GAF^132–364^), the histidine kinase plus the receiver domain (ETR1HKR^364–754^), the histidine kinase domain alone (ETR1HK^364–647^) and the receiver domain alone (ETR1R^647–754^), plus a cDNA encoding the NR receptor without the transmembrane domain (NR^117–635^), were cloned into the bait vector pEG202 (containing the DNA-binding domain of LexA, DB; Figure 2a,b). Partial cDNAs encoding the LeCTR2 N-terminus (CTR2N^50–700^) or C-terminus (CTR2C^680–982^) were inserted into the prey vector pJG4-5 (containing the activation domain, AD; Figure 2a,b). Tests for activation of reporter genes *LacZ* and *Leu2* by bait constructs alone in the absence of prey showed no activation of either reporter gene by any construct except the GAF domain (DB–ETR1^132–364^; Figure 2c, and data not shown). Synthesis of the bait proteins in yeast were confirmed by Western blotting using anti-LexA antibody (Figure 2d).

Three bait/prey combinations activated both *LacZ* (Figure 2e) and *LEU2* (data not shown) reporter genes, indicating that the N-terminus of LeCTR2 interacted with ETR1^132–754^, ETR1HKR^364–754^ and ETR1HK^364–647^; however, NR^117–635^/CTR2N^50–700^, ETR1R^647–754^/CTR2N^50–700^, ETR1^132–754^/CTR2C^680–982^ and NR^117–635^/CTR2C^680–982^ were unable to activate either reporter gene (Figure 2e, and data not shown). The GAF domain of LeETR1 could not be tested for interaction because of self-activation (Figure 2b,c), although LeETR1HKR^364–754^ and ETR1HK^364–647^, which lack the GAF domain, did show interactions. CTR2C^680–982^ did not interact with any region of LeETR1 or NR (Figure 2e, and data not shown). These results indicate an interaction between LeETR1 and LeCTR2 that requires the histidine kinase domain of LeETR1 and the N-terminus of LeCTR2, and no interaction between NR and LeCTR2. When the cDNAs encoding LeETR2, LeETR4, LeETR5 and LeETR6 without the transmembrane domains were cloned into pEG202 and tested for interactions with LeCTR2 in the yeast two-hybrid assay, only LeETR2 was able to interact with LeCTR2 (Figure S2 and Zhong *et al.*, 2008).

**Figure 3:**
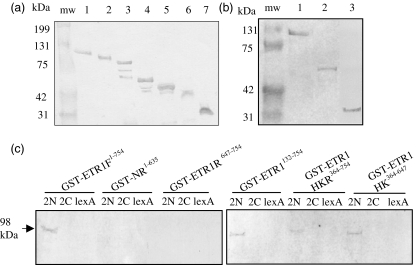
*In vitro* pull-down assays to test for interaction between various regions of LeETR1, NR and LeCTR2. (a) Western blotting to detect GST–receptor fusions using an anti-GST antibody. Lane 1, GST–ETR1F^1–754^; lane 2, GST–NR^1–635^; lane 3, GST–ETR1^132–754^; lane 4, GST–ETR1HKR^364–754^; lane 5, GST–ETR1HK^364–647^; lane 6, GST–ETR1R^647–754^; lane 7, GST control. mw, molecular weight markers. (b) Western blotting to detect DB–CTR2N^50–700^ (lane 1), DB–CTR2C^680–982^ (lane 2) and LexA control (lane 3) in yeast *S. cerevisiae* strain EGY48 using an anti-LexA antibody. mw, molecular weight markers. (c) Western blotting to detect DB–LeCTR2 fusions after incubation with GST–receptor fusions and pull-down using an anti-LexA antibody. 2N, DB–CTR2N^50–700^; 2C, DB–CTR2C^680–982^. The DB–CTR2N^50–700^ fusion protein was only detected (98 kDa, arrow) after incubation with GST–ETR1F^1–754^, GST–ETR1^132–754^, GST–ETR1HKR^364–754^ or GST–ETR1HK^364–647^.

The protein–protein interactions were also tested using *in vitro* pull-down assays. All the regions of LeETR1 used for interaction assays in yeast, together with the full-length LeETR1 and NR, were expressed as glutathione-*S*-transferase (GST) fusion proteins in *Schizosaccharomyces pombe* (Figure 3a and Figure S3a). Partial cDNAs encoding either CTR2N^50–700^ or CTR2C^680–982^ were cloned into pEG202 (DB–CTR2N^50–700^ and DB–CTR2C^680–982^; Figure S3b) and expressed in *Saccharomyces cerevisiae* (Figure 3b). For pull-down assays, purified GST–receptor fusions bound to the GST resin were incubated with yeast crude extract containing DB–CTR2N^50–700^, DB–CTR2C^680–982^ or LexA control (Experimental procedures). Immunoblotting using anti-LexA antibody indicated that DB–CTR2N^50–700^, with a molecular weight of 98 kDa (CTR2N^50–700^, 70 kDa; LexA, 28 kDa), was only detected in the lanes containing GST–ETR1F^1–754^/DB–CTR2N^50–700^, GST–ETR1^132–754^/DB–CTR2N^50–700^, GST–ETR1HKR^364–754^/DB–CTR2N^50–700^ and GST–ETR1HK^364–647^/DB–CTR2N^50–700^. No band was detected in the lanes containing GST–NR^1–635^/DB–CTR2N^50–700^ (Figure 3c).

### The C-terminus of LeCTR2 possesses kinase activity

**Figure 4:**
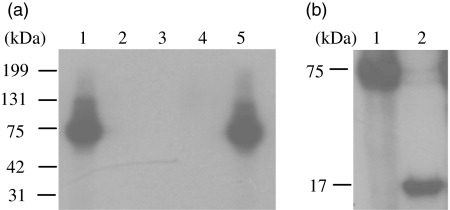
*In vitro* phosphorylation assay of various regions of LeCTR2. (a) Autophosphorylation of LeCTR2 N- or C-terminal domains. Lane 1, DB–CTR2N^50–700^; lane 2, DB–CTR2C^680–982^; lane 3, LexA; lane 4, DB–CTR2C^680–982^ plus staurosporine; lane 5, DB–CTR2C^680–982^ plus genstein. (b) Phosphorylation of myelin basic protein (MBP) by truncated DB–CTR2C^680–982^. Lane 1, DB–CTR2C^680–982^; lane 2, DB–CTR2C^680–982^ plus MBP.

To test whether LeCTR2 possesses kinase activity, DB–CTR2N^50–700^ and DB–CTR2C^680–982^ were immunoprecipitated from yeast using the anti-LexA antibody, and the purified fusion proteins together with LexA were incubated with [γ-^32^P]ATP, with or without kinase inhibitors and with myelin basic protein as a substrate. Radiolabelled phosphate was only incorporated into DB–CTR2C^682–982^ (Figure 4a, lane 2), not into DB–CTR2N^50–700^ or LexA (Figure 4a, lanes 1 and 3). The activity was abolished by the broad-spectrum protein kinase inhibitor staurosporine but the tyrosine kinase-specific inhibitor genstein had no effect (Figure 4a, lanes 4 and 5). DB–CTR2C^682–982^ was also able to phosphorylate myelin basic protein, a broad protein kinase substrate *in vitro* (Figure 4b, lane 2).

### Generation of LeCTR2 transgenic tomato plants

**Figure 5:**
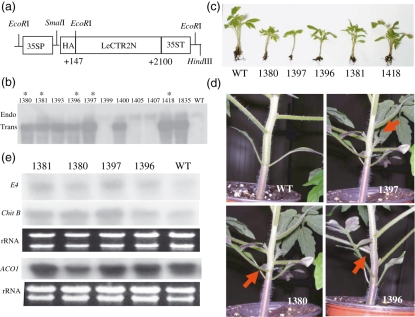
Characterization of transgenic plants overexpressing the LeCTR2 N-terminus. (a) Overexpression construct of the LeCTR2 N-terminus with cDNA nucleotide positions encoding amino acids 50–700 indicated. (b) Northern analysis of LeCTR2 primary transformants. Asterisks indicate the lines used in further studies. The *LeCTR2* cDNA (nucleotides 151–950), was used as a probe. Endo, endogenous LeCTR2 mRNA; Trans, transgene mRNA. (c) Reduced stature of the transgenic seedlings compared with the wild-type. Plants were grown in the soil and photographed at 14 days old. (d) The progeny of *LeCTR2* transgenic lines displayed earlier development of side shoots compared with the wild-type (arrows); photographs were taken at 34 days old. (e) Northern analysis of ethylene-related gene expression: 10 μg of total RNA from young leaves was probed with *E4* and *chitinase B* cDNAs, and 10 μg total RNA from mature green fruit was probed with *ACO1* cDNA. Ethidium-bromide stained rRNA indicates the sample loading.

The function of LeCTR2 in tomato was initially investigated using an antisense construct of *LeCTR2* cDNA from nucleotides 2134–2946, which covers the kinase domain, under the control of the 35S promoter (Figure S4a). Three independent lines containing the transgene were identified by Southern analysis (data not shown), but only one line partially suppressed the endogenous *LeCTR2* mRNA (Figure S4b). This line exhibited severe phenotypic effects, such as reduced stature (data not shown), excessive side shoots, prematurely senescing flowers, difficulty in fruit setting, and reduced trichomes (Figure S4c–e). Additional transformations with this construct failed to generate new plants, suggesting that downregulation of *LeCTR2* or closely related sequences was deleterious to plant growth. Accordingly, a partial cDNA of *LeCTR2* (nucleotides 147–2100), encoding the N-terminal regulatory domain, which has <40% similarity to other LeCTRs, was expressed in tomato under the control of the 35S promoter using *Agrobacterium tumefaciens*-mediated transformation (Figure 5a). Eleven primary transformants were regenerated and grown to maturity. Northern analysis identified eight lines resulting from independent transformation events that overexpressed the transgene (Figure 5b). Seeds collected from five lines (1380, 1381, 1396, 1397 and 1418) were grown to the next generation, and transgene inheritance and expression were confirmed by Northern and Southern analysis (Figure S5).

### Transgenic plants overexpressing the LeCTR2 N-terminus produced adventitious shoots and highly branched inflorescences

**Figure 6:**
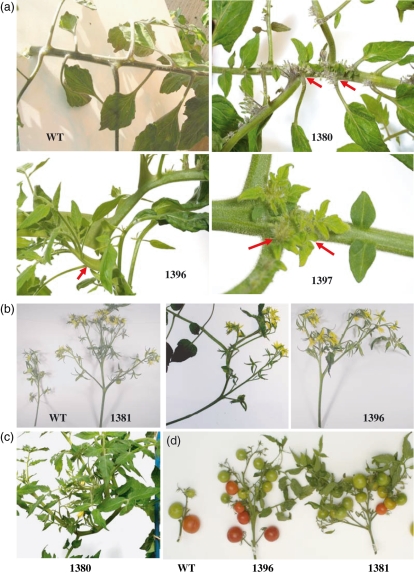
Phenotypes of transgenic plants overexpressing the LeCTR2 N-terminus. (a) Adventitious shoots arising from the rachis and rachillae of the leaves in transgenic lines 1380, 1396 and 1397 (arrows), but not in the wild-type. (b) Highly branched inflorescences and indeterminate shoots growing from determinate flowers in the transgenic lines, but not in the wild-type. The third inflorescence from each line was photographed. (c) Highly branched shoots developed from an inflorescence in line 1380. (d) Branched fruit trusses with abundant fruits from the transgenic plants compared with the wild-type. The second fruit truss for each plant was photographed.

During early development, transgenic lines overexpressing the LeCTR2 N-terminus were smaller compared with the wild-type (Figure 5c) and produced more side shoots. Transgenic plants at 34 days old had 3–5 side shoots compared with none in the wild-type (Figure 5d). Remarkably, prolific adventitious shoots were frequently found on the rachis or rachillae of the leaves of older plants (Figure 6a). The inflorescences were often highly branched, with indeterminate leaves in place of flowers (Figure 6b,c), and these sometimes developed into side shoots (Figure 6c). Flower numbers were often greater than the wild-type, and fruits were more abundant (Figure 6d), although there was no obvious effect on ripening (data not shown). Not all these phenotypes were seen in the primary transformants, but were pronounced in homozygous progeny and the characteristics co-segregated with the transgene construct (data not shown).

Measurement of ethylene production using six fully expanded young leaves from the same positions from 9-week-old transgenic and wild-type plants and 8-day-old light-grown seedlings showed that it was not significantly altered in the transgenic plants compared with wild-type (Table 1). Expression of the ethylene biosynthesis gene encoding ACC oxidase (*ACO1*; Hamilton *et al.*, 1990) was unaffected in the transgenic plants, whereas mRNA for the ethylene-responsive gene *E4* (Lincoln *et al.*, 1987) and *chitinase B* (*basic chitinase*; Danhash *et al.*, 1993) was more abundant, indicating enhanced ethylene signalling (Figure 5e).

**Table 1:** Ethylene production in LeCTR2 transgenic plants

Lines	Mature leaves (nl g^−1^ h^−1^)	Seedlings (nl g^−1^ h^−1^)
WT	3.76 ± 0.22	1.86 ± 0.26
1396–1	2.44 ± 0.07	1.87 ± 0.12
1397–1	2.94 ± 0.13	2.03 ± 0.34
1380–2	1.95 ± 0.03	1.48 ± 0.36
1381–1	3.09 ± 0.11	1.66 ± 0.12

Ethylene production was measured for both wild-type (WT) and transgenic lines overexpressing the LeCTR2 N-terminus. The data are the means of three measurements for each sample ± SEM.

### LeCTR2 transgenic plants exhibited enhanced susceptibility to the fungal pathogen Botrytis cinerea

**Figure 7:**
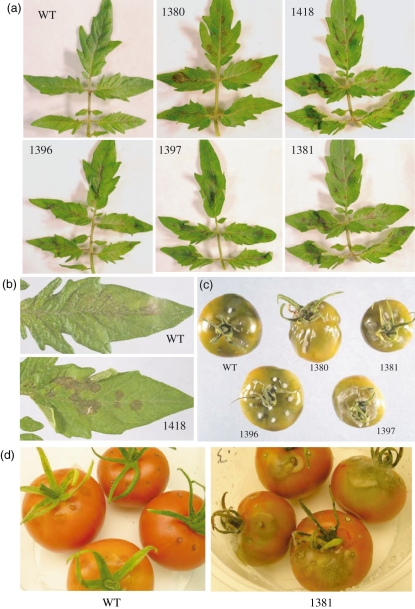
Response of wild-type and *LeCTR2* overexpressing plants to *B. cinerea* infection. (a) Transgenic leaves developed more and larger lesions than the wild-type leaves. Four compound leaves from each transgenic line and the control were detached from 5-week-old tomato plants and infected with 10 droplets of a 4 μl spore suspension containing 10^6^ spores ml^−1^, 0.01 m glucose and 6.7 mm KH_2_PO4. Photographs were taken 4 days after inoculation. Experiments were repeated twice. (b) Enlarged images from (a). (c, d) *B. cinerea* infection of fruits at the mature green (c) and ripening (d) stages from transgenic lines and the wild-type. Ten punctures on each fruit were made by a needle, and each wound was inoculated with a 4 μl *B. cinerea* spore suspension containing 10^6^ spores ml^−1^, 0.01 m glucose and 6.7 mm KH_2_PO_4_. Infection was evaluated and photographed 4 days after inoculation.

Leaves from the progeny of five independent transgenic lines and the wild-type were detached from 5-week-old plants and infected with *B. cinerea.* Four days after infection, the leaves from all transgenic lines showed a significant increase in disease symptoms compared with the control, with a considerable increase in lesion spreading (Figure 7a,b). To confirm this enhanced susceptibility, mature green and ripening fruits were also tested by inoculating puncture wounds with *B. cinerea*. Four days after inoculation, fruits from the transgenic lines displayed more severe infection, with a larger spreading area of soft rot and greater coverage of grey mould compared with the wild-type fruits, which showed limited disease development (Figure 7c,d).

**Figure 8:**
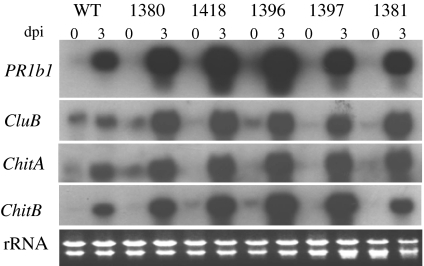
Northern analysis of pathogenesis-related gene expression in response to *B. cinerea* infection. Total RNA was isolated from the transgenic and wild-type leaves at 0 and 3 days after inoculation, and 10 μg was blotted and probed with the full-length cDNAs of *PR1b-1*, *Glucanase B* (*Gluc B*), and *Chitinases A* and *B* (*ChitA, ChitB*) *basic chitinases*. The ethidium-bromide stained rRNA indicates equal sample loading.

Analysis of pathogenesis-related (*PR*) gene expression three days after *B. cinerea* infection demonstrated that the transcripts of *PR1b1*, *Glucanase B* (*Gluc B*), and *Chitinase A* and *B* (*ChitA*, *ChitB*) were more highly induced compared with the wild-type (Figure 8).

## Discussion

LeCTR2 is a AtCTR1-like protein with a C-terminal serine/threonine kinase domain and the conserved N-terminal motifs (CN box) found in all AtCTR1-like proteins (Huang *et al.*, 2003). Sequence conservation suggests that these proteins have evolved from a common ancestor and may still have related functions. Our studies on enzymatic activity indicated that the C-terminus of LeCTR2 was able to autophosphorylate and phosphorylate a broadly used protein kinase substrate, myelin basic protein (Figure 4), suggesting that the protein probably participates in a phosphorylation cascade through the C-terminal kinase domain. Sequence comparison indicates that *LeCTR2* is more similar to Arabidopsis *EDR1* than to *AtCTR1*. The *LeCTR2* and *EDR1* genes are conserved with respect to the number, size and position of the exons, and the two proteins have 65% similarity, suggesting that they may play similar roles *in planta*.

Analysis of protein–protein interactions in the yeast two-hybrid system showed that LeCTR2 selectively interacts with two subfamily I ETR1-type receptors LeETR1 and LeETR2, but not with the subfamily I ERS-type receptor NR, or the subfamily II receptors (Figure 3 and Figure S2). The interaction of LeCTR2 with LeETR1, but not with NR, was confirmed by *in vitro* pull-down assays (Figure 3). Attempts to test an association between LeCTR2 and LeETR1 *in vivo*, using an anti-HA antibody to pull-down protein complex from extracts of *LeCTR2* transgenic plants were unsuccessful, possibly due to the presence of only a single copy of the haemagglutinin tag in the LeCTR2 construct.

Arabidopsis AtCTR1 has been shown to interact with both ETR1 and ERS1, the subfamily I receptors, in yeast (Clarke *et al.*, 1998), and this interaction is important for recruiting AtCTR1 to the ER and for signal transmission (Gao *et al.*, 2003). Recent studies have indicated that the *LeCTR* genes are not functionally identical. Although *LeCTR3* was able to complement the Arabidopsis *ctr1* mutant, *LeCTR1* and *LeCTR4* were only capable of weak or partial complementation (Adams-Phillips *et al.*, 2004), and the *ctr1* mutant could not be complemented using *LeCTR2* (S. Zhong, unpublished results). We have found that LeCTR1, LeCTR3 and LeCTR4 all interact with the subfamily I receptors LeETR1, LeETR2 and NR (Zhong *et al.*, 2008). Furthermore, each of these LeCTRs, but not LeCTR2, was recruited to the ER by NR. Domain deletions indicated that the LeETR1 histidine kinase (HK) domain alone was able to interact with the LeCTR2 N-terminus (Figures 3 and 4), suggesting that it is probably essential for the association with LeCTR2. The HK domain of Arabidopsis ETR1 has been shown to be required for signal transduction and serves as a signal output domain to AtCTR1 (Qu and Schaller, 2004). Sequence comparison of the HK domains of LeETR1, LeETR2 and NR indicates that LeETR1 is 88% identical to LeETR2, but only 61% identical to NR (data not shown). This variation may explain the failure of LeCTR2 to bind to NR, and suggests that LeETR1 and NR might interact with different signal output substrates. Although the receiver domain of the Arabidopsis ETR1 has been shown to be able to associate with the AtCTR1 N-terminus *in vitro* (Clarke *et al.*, 1998), two constructs of ETR1 – ETR1^364–754^ (with the receiver domain) and ETR1^364–647^ (without the receiver domain) – did not show much difference in interaction strength with LeCTR2 in our studies (Figure 2e), and the receiver domain alone (ETR1^647–754^) did not show any interactions with LeCTR2 either (Figures 2e and 3c).

Overexpression of the LeCTR2 N-terminus resulted in altered growth habit, including reduced stature and enhanced growth of side shoots even during early development (Figure 5). In older plants, adventitious shoots were frequently found on the rachis or rachillae of the leaves. In addition, highly branched inflorescences and fruit trusses were evident, and flower trusses often produced indeterminate shoots (Figure 6). Increased side shoot development and premature flower senescence were also found in a single antisense *LeCTR2* plant, in which *LeCTR2* expression was inhibited (Figure S4). There was no significant change in ethylene production by the transgenic plants, but there were higher levels of mRNA from the ethylene-responsive genes *E4* and *chitinase B* than in the wild-type, which suggests enhanced ethylene signalling (Figure 5e).

Liu *et al.* (2002) reported that silencing *LeCTR2* in tomato using virus-induced gene silencing (VIGS) did not produce any phenotypes in young plants, although VIGS of *LeCTR1* induced a constitutive ethylene response. In our studies, etiolated N-terminal LeCTR2 transgenic seedlings did not develop triple responses in the absence of ethylene (data not shown). Adult plants did not display the great reduction in size (see Figure 5c) found in *ctr1* mutants and the VIGS *LeCTR1* transgenic plants studied by Liu *et al.* (2002). In addition, the phenotypes we observed in response to LeCTR2 N-terminal expression were more pronounced in homozygous progeny, particularly in mature plants, which were not studied by Liu *et al.* (2002).

Transgenic plants overexpressing the LeCTR2 N-terminus also displayed enhanced susceptibility to the fungal pathogen *B. cinerea* (Figure 7), and this was associated with stronger accumulation of a number of *PR* gene transcripts (Figure 8). Ethylene is implicated in biotic stress as a virulence factor of fungal and bacterial pathogens and as a signalling compound in disease resistance, and ethylene treatment typically promotes *B. cinerea* disease development (Van Loon *et al.*, 2006). In some cases, inhibiting ethylene synthesis or perception has been reported to reduce susceptibility to pathogen infection, although this is not always consistent (Van Loon *et al.*, 2006). For example, Cooper *et al.* (1998) showed that infection of transgenic fruits in which the ethylene biosynthesis gene encoding ACC oxidase (ACO) had been inhibited by an antisense gene progressed more slowly in response to the post-harvest pathogen *Colletotrichum gleoeosporioides* compared with wild-type. Furthermore, the tomato ethylene receptor mutant *Never-ripe* (*Nr*), which is insensitive to ethylene, showed reduced disease symptoms compared with the wild-type after infection with bacterial (*Xanthomonas campestris* pv. *vesicatoria* and *Pseudomonas syringae* pv. *tomato*) and fungal (*Fusarium oxysporum* f. sp. *lycopersici*) pathogens (Lund *et al.*, 1998).

Our results strongly suggest that LeCTR2 plays a different role from LeCTR1, but the protein–protein interaction evidence (Figures 2 and 3), enhanced *E4* and *chitinase B* mRNA levels (Figure 5) and greater susceptibility to pathogen infection (Figure 7) do support a role for LeCTR2 in ethylene signalling. Further studies, however, are necessary to test whether the association between LeCTR2 and LeETR1/2 occurs *in planta*. The selective interactions of LeCTR2 with LeETR1 and LeETR2, but no other receptors, suggests that LeCTR2 might function in a specific branch of ethylene signalling. The excessive side-shoot growth, adventitious shoot formation, and highly branched inflorescences of *LeCTR2* transgenic plants are consistent with reduced auxin and enhanced cytokinin responses, and this could result from enhanced ethylene signalling. The phenotype of the transgenic plants could be explained if the truncated LeCTR2 N-terminus competes or interferes with the wild-type LeCTR2 protein for binding to the ethylene receptor, thereby interfering with the normal LeCTR2 function *in vivo* and producing a dominant-negative phenotype, as found for overexpression of the AtCTR1 N-terminus (Huang *et al.*, 2003). It is also possible, however, that overexpression of the LeCTR2 N-terminus could interfere with the association of other CTRs with ethylene receptors, thereby preventing their normal functions, as LeETR1 and LeETR2 can also interact with LeCTR1, LeCTR3 and LeCTR4 in yeast two-hybrid assays (Zhong *et al.*, 2008).

EDR1 was identified by screening disease resistant mutants, and was initially proposed to function at the head of a MAP kinase cascade that negatively regulates salicylic acid-dependent defence responses (Frye *et al.*, 2001). More recently, its effects on ethylene-related senescence and cell death led to the suggestion that it functions at a point of cross-talk between ethylene and salicylic acid signalling (Tang *et al.*, 2005), although the upstream signalling components, including possible physical associations with ethylene receptors, are unknown. In this study, we have demonstrated that LeCTR2 has a role in response to disease and is also implicated in ethylene signalling, indicating a clear link between these processes.

## Experimental procedures

### Generation of constructs and transgenic plants

All molecular cloning procedures were carried out using standard methods (Sambrook *et al.*, 1989). The *LeCTR2* cDNA from nucleotide +147 to +2100 with a single copy of the haemagglutinin (HA) sequence at the 5′ end was amplified by PCR and inserted into pDH51. This insertion resulted in positioning of the *LeCTR2* fragment in the sense orientation with respect to the CaMV 35S promoter and the terminator in pDH51. pDH51 containing the transgene was then cut using *Eco* RI and inserted into pBin19 (Bevan, 1984). The resulting construct was introduced into competent *Agrobacterium tumefaciens* LB4404 cells (Bevan, 1984) and used to transform wild-type cotyledon cells of tomato (*Solanum esculentum* L. cv. Ailsa Craig). Plantlets were generated on 100 μg ml^−1^ kanamycin and transferred to compost. Transformants and wild-type controls were grown under standard greenhouse conditions.

### RNA isolation and Northern analysis

RNA extraction and blotting were carried out as described by Griffiths *et al.* (1999). Hybridizations were carried out for 16 h at 42°C in buffer containing 1% w/v SDS, 50% v/v deionized formamide, 5× SSC, 50 mm sodium phosphate pH 6.8, 0.1% w/v sodium pyrophosphate, 10% w/v dextran sulfate and 50 μg ml^−1^ salmon sperm DNA. Radiolabelled probes were prepared using the Rediprime II random prime labelling system (Amersham Pharmacia Biotech, http://www5.amershambiosciences.com/). Hybridized membranes were washed in 0.2 × SSC, 0.1% SDS and autoradiography was used to detect the signal.

### Genomic DNA isolation and Southern blot

Genomic DNA was extracted using a GenElute plant genomic DNA miniprep kit according to the manufacturer's instructions (Sigma, http://www.sigmaaldrich.com/). Individual genomic DNA samples (10 μg) were completely digested with *Eco* RI, separated on a 0.8% agarose gel, and capillary blotted to Gene Screen membrane (Perkin Elmer, http://las.perkinelmer.co.uk). Hybridization was carried out using the procedure outlined above.

### Yeast two-hybrid analysis

The LexA-based interaction trap system described by Golemis and Brent (1997) was used in this study. All plasmids and *S. cerevisiae* strain EGY48 were kindly supplied by R. Brent (Massachusetts General Hospital, Boston, MA, USA). ‘Bait’ proteins consisting of partial ethylene receptor sequences and various domains of LeETR1 were constructed by insertion of cDNA sequences into the *Eco* RI/*Xho* I or *Bam* HI/*Xho* I restriction sites of plasmid pEG202, downstream of and in-frame with the bacterial LexA DNA-binding domain sequence (DB), producing DB–ETR1^132–754^, DB–ETR1HKR^364–754^, DB–ETR1HK^364–647^, DB–ETR1R^647–754^, DB–ETR1GAF^132–364^ and DB–NR^117–635^, respectively. All the constructs were confirmed by sequencing. The homeodomain of bicoid protein fused to the LexA DNA-binding domain, encoded in plasmid pRFHM1, was used as a negative control, and pSH17-4 encoding the LexA DNA-binding domain upstream of the Gal4 activation domain was used as a positive control. ‘Prey’ proteins consisting of the LeCTR2 N-terminal region (CTR2N^50–700^) or C-terminal region (CTR2C^680–982^) were prepared by insertion of PCR-amplified cDNA sequences into the *Eco* RI/*Xho* I restriction sites of pJG4-5, downstream of the activation domain of the acid blob B42 (AD).

The cDNAs encoding LeETR2, LeETR4, LeETR5 and LeETR6 without the transmembrane domains were PCR-amplified and cloned into the bait vector pEG202 to form constructs DB–ETR2^115–732,^ DB–ETR4^140–761^, DB–ETR5^140–747^ and DB–ETR6^146–734^, respectively (Figure S2a). All the constructs were sequenced.

### Preparation of GST fusion proteins and in vitro pull-down assay

The cDNAs encoding the full-length LeETR1 protein (amino acids 1–754) and various deletions used in yeast two-hybrid assays, and the cDNA encoding the full-length NR protein, were amplified by PCR, and inserted into the *Bam* HI site of vector pESP-2 (Stratagene, http://www.stratagene.com/) in-frame with the GST tag. Constructs were confirmed by sequencing and then transformed into yeast *Saccharomyces pombe* strain SP-Q01. Total proteins were extracted in PBST containing proteinase inhibitors (140 mm NaCl, 2.7 mm KCl, 10 mm Na_2_HPO_4_, 1.8 mm KH_2_PO_4_, 1% Triton® X-100, 1 mm PMSF and 100 μm leupeptin). GST fusion proteins were purified on GST affinity resin according to the manufacturer's instructions (Stratagene), and then visualized by Coomassie blue staining (CBB R250) and verified using an anti-GST antibody (Amersham). Partial cDNAs encoding the LeCTR2 N-terminus (CTR2N^50–700^) or the C-terminus (CTR2C^680–982^) were inserted into pEG202 to form DB–CTR2N^50–700^ and DB–CTR2C^680–982^. These constructs were introduced into yeast *S. cerevisiae* strain EGY48 and grown in minimal medium lacking histidine (Golemis and Brent, 1997) at 29°C overnight. Total proteins were extracted in PBST plus proteinase inhibitors as described above, and quantified using the Bio-Rad protein assay (http://www.bio-rad.com/). Expression of the LexA fusion proteins was detected using an anti-LexA antibody (Invitrogen) after immunoblotting. For *in vitro* pull-down assays, 1 μg of each purified GST–receptor fusion protein was bound to GST affinity resin, and 200 μg of total yeast extracts containing DB–CTR2N^50–700^, DB–CTR2C^680–982^ or the control vector were added. Interaction samples were maintained in 1 ml of sucrose buffer (100 mm Tris pH 7.5, 0.3 m sucrose, 5 mm EDTA, 2 mm PMSF, 1 μm leupeptin) and incubated with rotation for 1 h at 4°C. After washing three times in PBST, samples were subjected to SDS–PAGE (8%), and complexes were detected using an anti-LexA antibody.

### Immunoprecipitation and kinase assay

DB–CTR2N^50–700^, DB–CTR2C^680–982^ or LexA were purified from *S. cerevisiae* by immunoprecipitation using an anti-LexA antibody. Total yeast cell extract (200 μg) was bound to 50 μl of LexA antibody–protein A–Sepharose in 1 ml of ice-cold immuno-precipitation buffer and incubated with rotation for 1 h at 4°C. Samples were washed three times in PBST prior to the kinase assay. The purified protein with or without protein kinase inhibitors staurosporine (125 nm) and genstein (100 nm; Sigma) was incubated for 30 min at 30°C in 20 μl of kinase buffer (50 mm Tris pH 7.8, 50 mm KCl, 2 mm DTT, 5 mm MnCl_2_, 10% glycerol) and 2 μCi [^32^P]-γATP. For the trans-phosphorylation assay, 1 μg myelin basic protein was added to each assay. The reactions were terminated with 2× SDS sample buffer. Samples were subjected to 8% SDS–PAGE, and fixed, dried and exposed to X-ray film at −70°C.

### Measurement of ethylene production from leaves and seedlings

Ethylene was measured according to the method described by Smith *et al.* (1986). Six leaves or seedlings were weighed, placed in a 25 ml glass bottle and sealed using ‘Subaseal’ vaccine caps (Scientific Laboratory Supplies, http://scientificlabs.eu). After 2 h, 1 ml of gas from the headspace was withdrawn and ethylene was analysed on a gas chromatography apparatus (Pye Unicam, http://unicam.co.uk). The results are expressed as nl g^−1^ h^−1^.

### B. cinerea infection

*B. cinerea* infection was carried out according to the method described by Audenaert *et al.* (2002) with slight modification. *B. cinerea* was grown on potato agar (under a light regime of 12 h UV/12 h dark). After 10 days, spores were washed from the plates using distilled water containing 0.01% v/v Tween-20. After removing mycelial debris, spores were counted and added to the inoculation solution (0.01 m glucose, 6.7 mm KH_2_PO_4_) at 10^6^ spores ml^−1^.

Five compound leaves from each *LeCTR2* transgenic line and the wild-type were detached from 5-week-old tomato plants, placed in 125 mm plastic pots containing 8% agar, and infected with 10 droplets of 4 μl spore suspension (described above). Symptoms were examined daily. Four fruits from each *LeCTR2* transgenic line and wild-type at the breaker stage (start of colour change) and three days after the start of colour change were detached and placed in 125 mm pots containing wet Whatman 3 mm paper (Schleicher and Schuell, http://www.farnell.co.uk). Ten puncture wounds for each individual fruit were made using a 0.5 × 25 mm needle, and 4 μl of inoculation solution (described above) were applied to each wound. Symptoms were examined daily.

## Supplementary material

The following supplementary material is available for this article online:

Figure S1 Sequence analysis of LeCTR2. (a) Genomic structure comparison of LeCTR2 and EDR1. Exons are depicted as white boxes and introns as variable sized wedges in proportion to the size of the intron. Regions upstream of the start codon and downstream of the stop codon are represented as black boxes. (b) Phylogenetic tree of LeCTR2, LeCTR1, LeCTR3, LeCTR4, *Arabidopsis* CTR1 and EDR1 using the N-terminal regions of these proteins. The branch length was scaled.

Figure S2 Protein-protein interaction assays between LeCTR2 and receptors LeETR2, LeETR4, LeETR5 and LeETR6. (a) Constructs of the bait proteins. Partial cDNAs encoding the ethylene receptors LeETR2, LeETR4, LeETR5, and LeETR6 without the transmembrane domain were inserted downstream of the LexA DNA-binding domain (DB) in the bait vector pEG202. Numbers indicate amino acid residues. Domains are indicated with featured boxes. (b) Yeast expressing bait proteins in the absence of the prey constructs was tested for activation of the *LacZ* reporter gene by incubation on minimal medium containing glucose (Glu) and X-gal. (c) Western blot to determine the bait protein synthesis in yeast using an anti-LexA antibody. R2: DB-ETR2^115−732^; R4: DB-ETR4^140−761^; R5: DB-ETR5^140−747^; R6: DB-ETR6^146−734^. (d) Activation of the *LacZ* reporter gene by protein-protein interactions between LeCTR2 and receptors. Only yeast containing DB-ETR2^115−732^/AD-CTR2N^50−700^(R2/N2) turned dark blue within three days on plates containing galactose and x-gal (gala/x-gal). Yeast containing other receptor constructs and LeCTR2-N remained white on gala/x-gal plates. R2: DB-ETR2^115−732^; R4: DB-ETR4^140−761^; R5: DB-ETR5^140−747^; R6: DB-ETR6^146−734^; 2N: AD-CTR2N^50−700^; P: Positive control; N: Negative control.

Figure S3 Constructs for *in vitro* pull-down interaction assays. (a) The full-length LeETR1 and NR, and partial LeETR1 sequences were inserted into pESP-2 in frame with the GST tag. Amino acid residues are numbered for each construct. (b) The cDNAs encoding the N- or C- terminus of LeCTR2 were cloned into pEG202 downstream of the LexA DNA binding domain (DB). Amino acid residues are indicated.

Figure S4 Characterisation of the LeCTR2 antisense silencing line. (a) Antisense construct of LeCTR2 with cDNA nucleotide positions. (b) Northern analysis of endogenous LeCTR2 expression in the LeCTR2 antisense line using 30 μg total RNA from 14-days-old seedlings and a partial cDNA of LeCTR2 as a probe (nt: 1––900). Ethidium bromide stained rRNA indicates equal sample loading. (c-e), Phenotypes caused by inhibiting LeCTR2 expression. Excessive side shoot growth (c); Premature flower senescence (d); Greatly reduced trichomes (e).

Figure S5 Characterisation of the transgene inheritance in the progeny of LeCTR2 N-terminal overexpressors. (a) Southern analysis of LeCTR2 progeny with neomycin phosphotransferase gene *NPTII* as a probe. 10 μg genomic DNA from each plant was digested with *Eco* RI and separated in 1% agarose gel before blotting. The lines 1380, 1381, 1396, 1373, and 1384 are the same as those asterisked in Figure 5 (b). (b) Northern analysis of LeCTR2 progeny: 30 μg total RNA from young leaves of germinated seedlings was probed with the *LeCTR2* cDNA (nt 150-950). Endo: endogenous LeCTR2 mRNA; Trans: transgene LeCTR2N mRNA.
