# Supplementary material for: LeCTR2, a CTR1-like protein kinase from tomato, plays a role in ethylene signalling, development and defence
Source: Plant J. 2008 Apr 25;54(6):1083–93. doi: 10.1111/j.1365-313X.2008.03481.x (PMC2440563; doi:10.1111/j.1365-313X.2008.03481.x)
Supplement: Figure S1 — Sequence analysis of LeCTR2. (a) Genomic structure comparison of LeCTR2 and EDR1. Exons are depicted as white boxes and introns as variable sized wedges in proportion to the size of the intron. Regions upstream of the start codon and downstream of the stop codon are represented as black boxes. (b) Phylogenetic tree of LeCTR2, LeCTR1, LeCTR3, LeCTR4, Arabidopsis CTR1 and EDR1 using the N-terminal regions of these proteins. The branch length was scaled. [file tpj0054-1083-SD1.pdf]

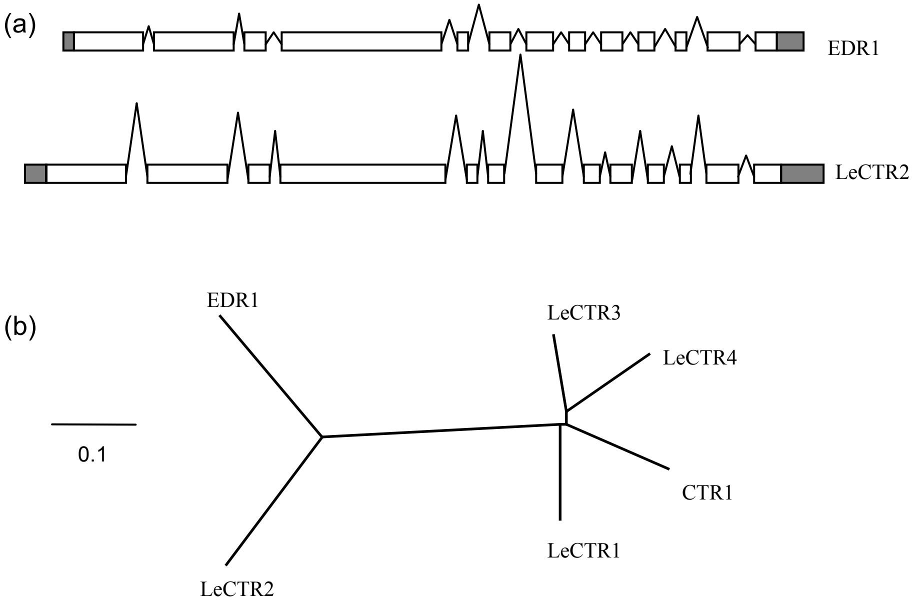

Supplementary Figure 1

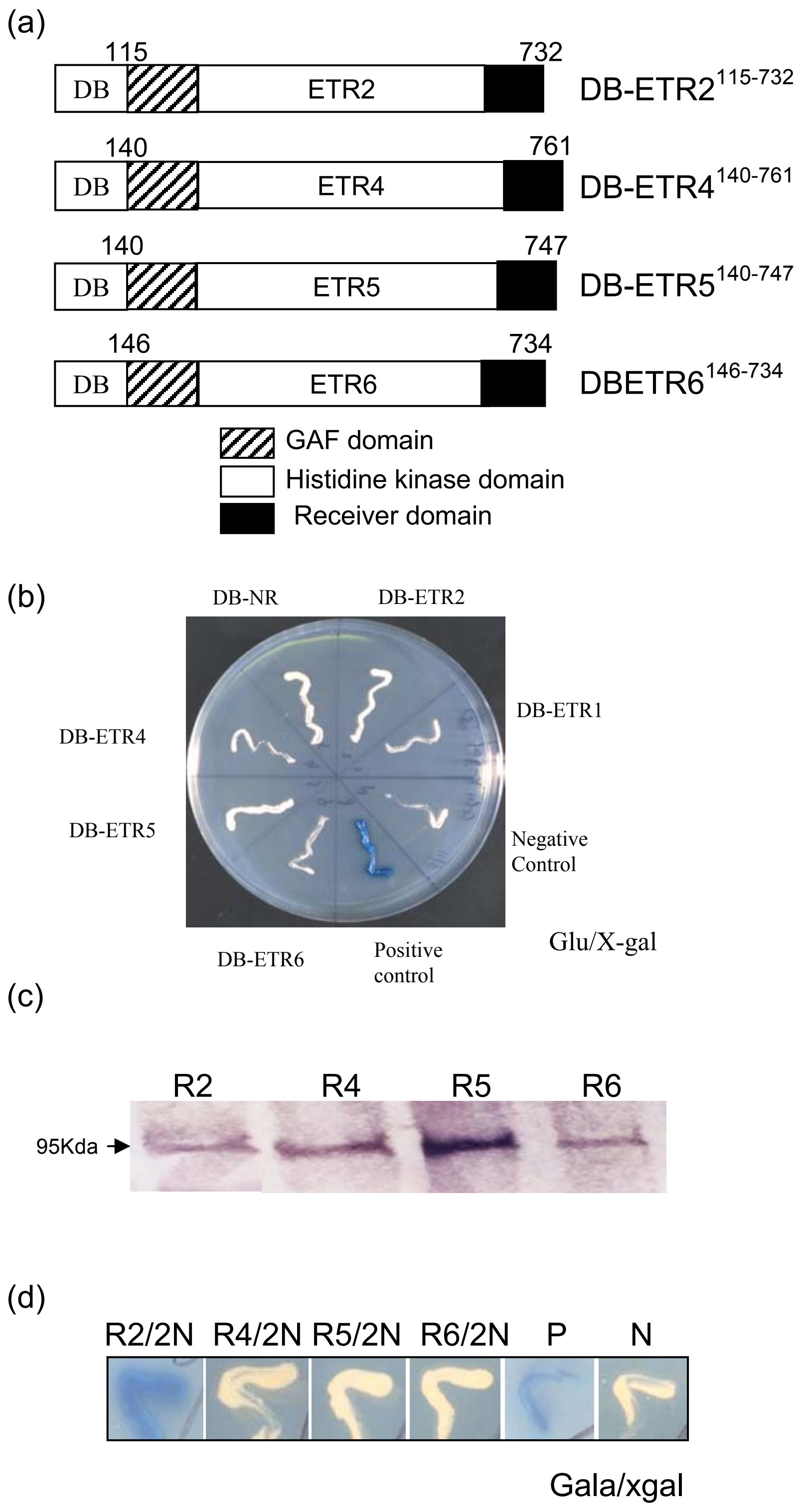

Supplementary Figure 2

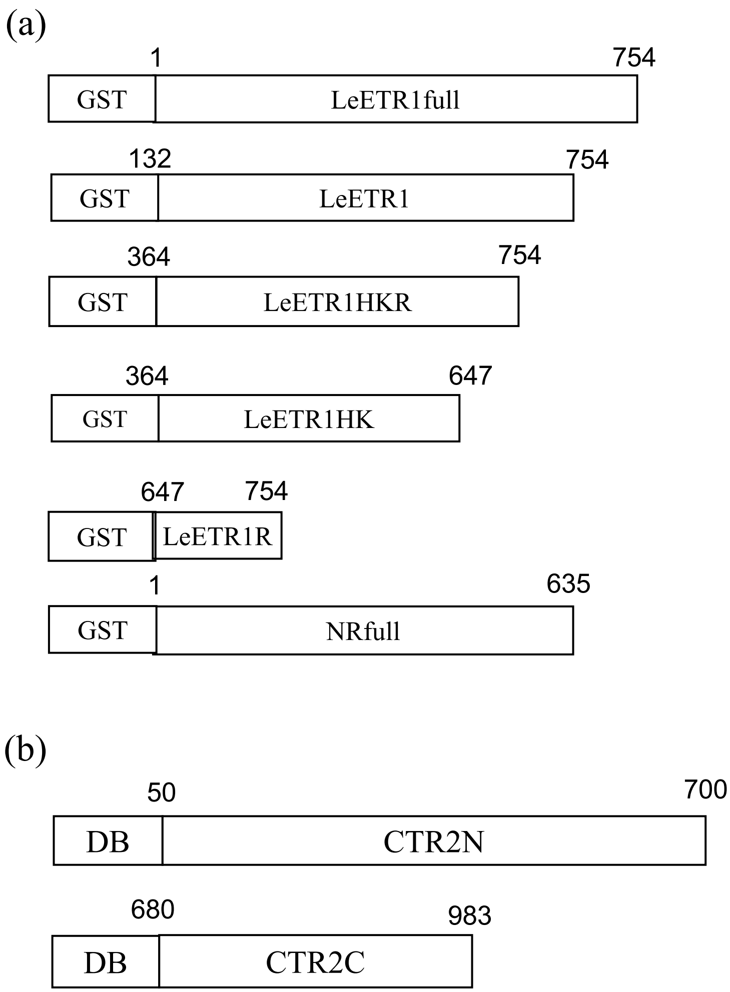

Supplementary Figure 3

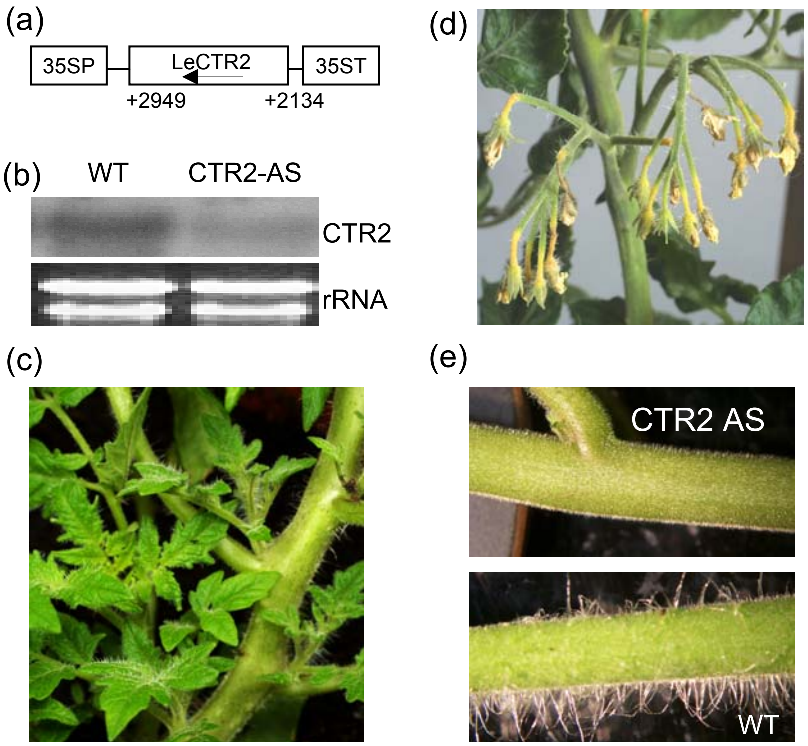

Supplementary Figure 4

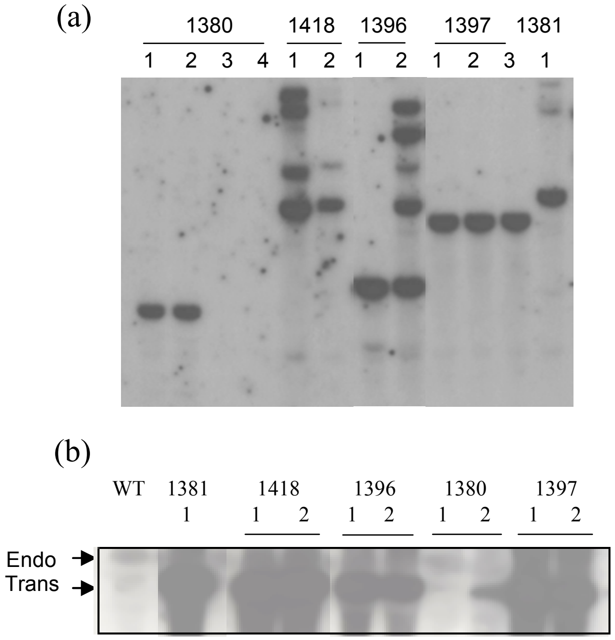

Supplementary Figure 5
